# Plasma phenoloxidase of the larval tobacco budworm, *Heliothis virescens,* is virucidal

**DOI:** 10.1673/2006_06_13.1

**Published:** 2006-08-10

**Authors:** Kent S. Shelby, Holly J. R. Popham

**Affiliations:** USDA, Agricultural Research Service, Biological Control of Insects Research Laboratory, 1503 S. Providence Rd., Columbia, MO 65203, United States

**Keywords:** catalase, hydrogen peroxide, HzAM-1 cells, HzSNPV, Kojic acid, nitric oxide synthase, superoxide, superoxide dismutase, HzSNPV, Helicoverpa zea single nucleopolyhedrosis virus, HvPPO, Heliothis virescens prophenoloxidase, HvPO, Heliothis virescens phenoloxidase, PTIO, 2-phenyl-4,4,5,5-tetramethylimidazoline-1-oxyl 3 oxide

## Abstract

Heliothis virescens larval plasma contains high levels of an antiviral activity against the budded form of the Helicoverpa zea single nucleopolyhedrovirus (*Hz*SNPV) *in vitro*. Preliminary results indicated that phenoloxidase is primarily responsible for this virucidal effect. However it is known that other enzymes that generate antimicrobial reactive oxygen intermediates and reactive nitrogen intermediates are present in hemolymph that could contribute to the observed virucidal activity. To elucidate the contributions of phenoloxidase and other candidate activities to plasma innate immune response against baculovirus infection specific metabolic inhibitors were used. *In vitro* the general inhibitors of melanization (N-acetyl cysteine, ascorbate and glutathione), and specific inhibitors of phenoloxidase (phenylthiourea and Kojic acid), completely blocked virucidal activity up to the level seen in controls. Addition of the enzyme superoxide dismutase to plasma did not affect virucidal activity; however addition of catalase had an inhibitory effect. Inhibitors of nitric oxide synthase activity did not affect virucidal activity. Our results confirm that phenoloxidase is the predominate activity in larval plasma accounting for inactivation of *Hz* SNPV *in vitro*, and that phenoloxidase-dependent H_2_O_2_ production may contribute to this virucidal activity.

## INTRODUCTION

Insects possess some of the innate antiviral immune responses homologous to those present in vertebrates. For example, Drosophila melanogaster may utilize the mechanism of RNA interference to resist infection by the flock house virus (Li *et al.* 2002). Recently it has been reported that recognition of an infection caused by Drosophila X virus in Drosophila larvae may be mediated by a toll receptor (Zambon *et al.* 2005). The plasma protein hemolin is induced by baculovirus infection of the Chinese oak silkmoth, Antheraea pernyi, and other lepidoptera (Hirai *et al.* 2004). Infection with the tomato spotted wilt virus of its vector insect, the western flower thrip, Frankliella occidentalis, resulted in upregulation of a suite of antimicrobial response genes homologous to those identified in other insects (Medeiros *et al.* 2004). The primary mechanism of lepidopteran larval resistance to *per os* baculovirus infection is clearing of infected midgut cells by apoptosis (Clarke and Clem 2003). Cell-mediated antiviral immune mechanisms utilizing melanization of infective foci have also been observed in the hemocoel of infected larvae, *i.e.* melanotic encapsulation of AcMNPV infected midgut and tracheoblast cells by corn earworm,Helicoverpa zea, hemocytes (Washburn *et al.* 1996;Washburn*et al.* 2000;Trudeau *et al.* 2001).

Beyond the infective foci of the midgut and tracheoblasts, levels of the plasma enzyme phenoloxidase [PO: L-DOPA: oxygen oxidoreductase; EC 1.14.18.1] in larval lepidoptera are correlated with resistance to baculovirus infection (Wilson *et al.* 2001). Plasma phenoloxidase (HvPO) of the tobacco budworm, Heliothis virescens (F.), has been demonstrated to exhibit *in vitro* a virucidal activity against several vertebrate viruses (Ourth and Renis 1993; Ourth 2004) and against the budded form of the baculovirus H. zea single nucleopolyhedrosis virus (HzSNPV) (Popham *et al.* 2004). In this report further evidence is provided for inactivation of baculovirus budded virus particles by phenoloxidase in isolated plasma *via* phenoloxidase-dependent production of reactive oxygen species.

## MATERIALS AND METHODS

### Chemicals

Chemicals, enzymes and inhibitors were purchased from Sigma Chemical Co. (www.sigmaaldrich.com).

### Insects, and insect diets

H. virescens eggs were received from the North Carolina State University Dept. of Entomology Insectary from a colony established from field insects in July of 2002. Larvae were reared individually on an artificial wheat germ based diet (Catalog # F9781B, BioServe, www.bio-serv.com) under a photoperiod of 14:10 L:D at 55% relative humidity at 28°C to the appropriate assay instar. This diet contained a basal level of ascorbic acid of 2.4 g/L (personal communication, BioServe technical staff). Diets with elevated levels of ascorbic acid were prepared by adding ascorbic acid in multiples of 2.4 g/L to the basal amount during mixing. Pupation and emergence times were determined by the ViStat 2.1 program (Hughes 1990).

### Insect cells and virus

An H. zea cell line, *Hz*AM-1, was maintained as monolayers at 28°C in Excel 401 medium (JRH Biosciences, www.jrhbio.com) supplemented with 10% fetal bovine serum (Integen Co., www.intergenco.com). Wild type *Hz*SNPV isolate was used and amplified in *Hz* AM-1 cells (Popham *et al.* 2004).

### Collection and Processing of Plasma

Staged larvae were surface sterilized in ethanol, rinsed with sterile water, and anesthetized on ice before bleeding. Hemolymph was gently extruded from an anterior proleg, through a small puncture wound made with a sterile 26 gauge needle, and collected directly into a chilled 1.5 ml microcentrifuge tube containing ice cold, sterile PBS (50 mM NaHPO_4_, pH 6.8) (Popham *et al.* 2004). Hemolymph was adjusted to a final dilution of 1:10 by addition of cold PBS after which hemocytes were removed by centrifugation at 8,000 rpm for 3 minutes. The plasma supernatant was sterilized by centrifugation through a 0.65 micron Millipore Ultrafree™-MC centrifugal filter (Millipore, Inc., www.waters.com). Aliquots of each filter sterilized plasma collection were allowed to stand at room temperature for two hours to individually monitor the extent of melanization. When used, inhibitors were added to buffer before hemolymph collection at the concentrations indicated.

### Plasma *in vitro* virucidal assay

Virucidal activity in larval H. virescens plasma was quantified by endpoint dilution assay as detailed (Popham *et al.* 2004). In brief, plasma dilutions were combined with *Hz* SNPV at a ratio of 3:1 (v/v) and allowed to incubate at 20°C for 1 h. PBS was used as a control in the absence of plasma. Viral titers of these incubations were determined by end-point dilution assay (Slavicek *et al.* 2001). *Hz*AM-1 cells were seeded at 5 x 10^4^ cells/ml in P96 well plates (BD Falcon, www.bdbiosciences.com) and allowed to attach for 1 h. The cells were infected with dilutions of virus/plasma or virus/PBS at dilutions of 10^−1^ to 10^−6^ and plates were incubated for one week at 28°C. The plate wells were then scored positive, if polyhedra were visible within two or more cells, or negative for viral infection, and the results were used to calculate the viral titer as the tissue culture infectious dose per ml (TCID_50_/ml) of inoculum. Plasma samples were assayed in quadruplicate. Statistical comparisons were done using SigmaStat (SPSS Inc.,www.spss.com).

### Phenoloxidase activity assay

HvPO activity was measured according to a modification of Jiang *et al.* 2003 using dopamine as a substrate. Twenty μl of diluted plasma was mixed with 800 μl of 2 mM dopamine in 50 mM sodium phosphate, pH 6.5, and the change in absorbance at 472 nm was then monitored using a BioMate-3 spectrophotometer (ThermoSpectronic, Rochester, NY) in triplicate.

## RESULTS

Virucidal activity in H. virescens larval plasma was measured throughout development from the late 3^rd^ instar until the late 5^th^ instar (Table 1). High activity, two orders of magnitude reduction in viral survival, was evident from the late 3^rd^ until the early 5^th^ instar, but declined almost to control PBS buffer levels as 5^th^ instar development proceeded (data not shown). For this reason, and because larger volumes of plasma could be more easily collected, early 5^th^ instar larvae were bled for all experiments shown below.

**Table 1.**
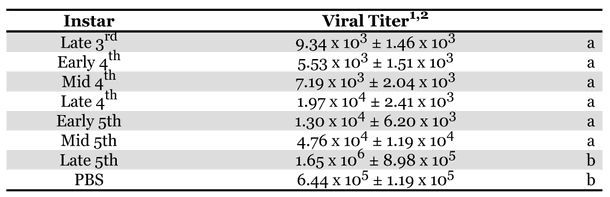
Plasma antiviral activity against HzSNPV over the course of the 3rd, 4th, and 5th larval instars of H. virescens.

**Figure 1.**
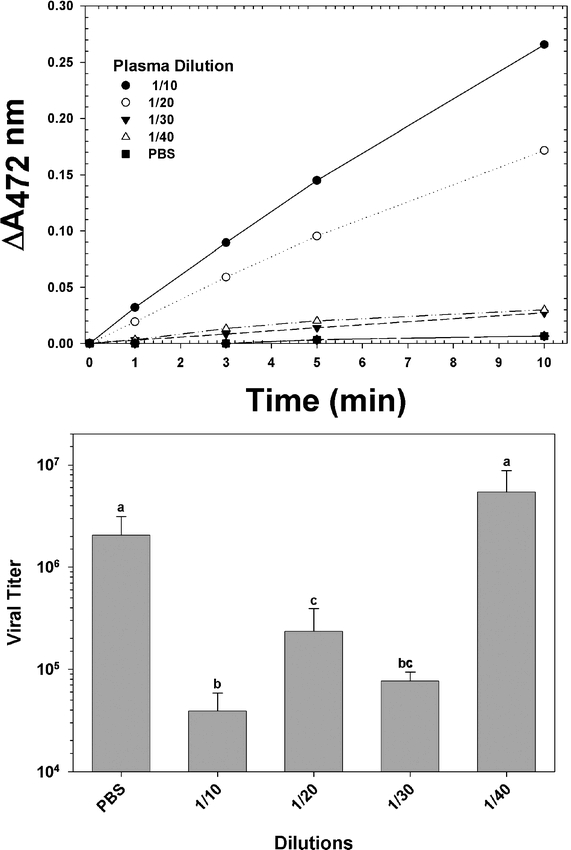
Correlations between phenoloxidase and virucidal activity in early 5^th^ instar larval Heliothis virescens plasma dilutions. (A) Spectrophotometric determination of phenoloxidase activity in serial dilutions of plasma. PBS, phosphate buffered saline. (B) Virucidal activity determined from the same fractions (n = 3, mean ± SEM).

If virucidal activity were dependent upon the activity of HvPO, then the two activities should be strongly correlated. We therefore measured plasma HvPO activity according to Jiang *et al* 2003, and virucidal activity in the same plasma fractions (Figs. 1A and 1B). High HvPO activity was observed in all 1/10 diluted plasma samples; the optimum dilution empirically determined for virucidal activity assays (Popham *et al.*, 2004). HvPO enzymatic activity was rapidly diluted by the addition of PBS (n = 4, p < 0.02, Student-Newman-Keuls multiple comparison test; Fig. 1A). HvPO activity was not evident at dilutions below 1/30 during the 10 minute incubations (Fig 1A). Virucidal activity in the same fractions also was rapidly diluted, becoming undetectable at the 1/40 dilution in the standard one hour incubation period (n = 4, p < 0.001, Tukey multiple comparison test; Fig. 1B), *c.f.*Popham *et al.* 2004.

**Figure 2.**
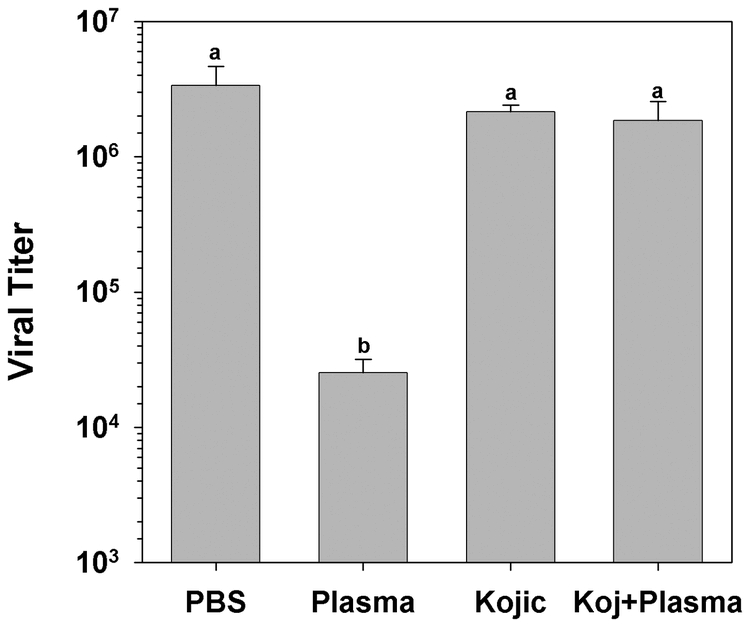
The effect of specific phenoloxidase inhibitors on larval Heliothis virescens plasma virucidal activity. The inhibitors were added to diluted plasma samples before the addition of HzSNPV. PBS, No plasma control; Kojic, 1 mM Kojic acid control; Kojic + Plasma, 1 mM Kojic acid added to plasma (letters indicate significant differences between treatments; n = 4, mean ± SEM).

Addition of serine proteinase inhibitors, that prevent the activation of HvPPO to HvPO, and of the inhibitor phenylthiourea, that chelates copper from the active site of the enzyme, was previously demonstrated to inhibit plasma virucidal activity in larval H. virescens plasma (Popham *et al.* 2004). To further elucidate the enzymatic nature of the virucidal activity, additional specific inhibitors of HvPO activity were sought. Kojic acid (5-hydroxy-2-hydroxymethyl-γ-pyrone) is a specific competitive inhibitor of insect phenoloxidases (Dowd 1988; Chen *et al.* 1991a; Chen *et al.* 1991b; Li and Kubo 2004). Preincubation of diluted early 5^th^ instar H. virescens plasma with 1 mM Kojic acid completely abolished virucidal activity (n = 4, p < 0.013, Student-Newman-Keuls multiple comparison test; Fig. 2). Additionally, the copper chelator diethyldithiocarbamic acid when fed to larvae has been reported to suppress resistance to infection with the baculovirus AcMNPV (Washburn *et al.* 1996). This inhibitor proved to be too toxic to *Hz*AM-1 cells and thus could not used in further experiments (data not shown).

**Figure 3.**
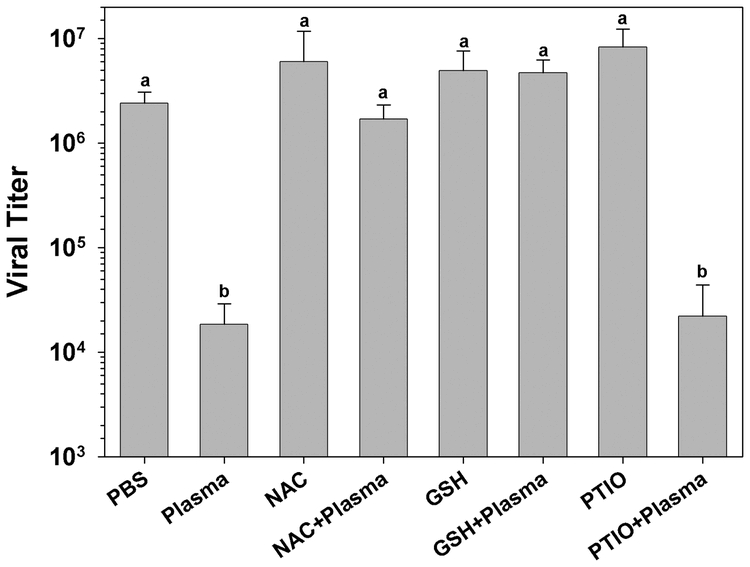
The effect of the free radical scavengers N-acetyl cysteine (NAC), reduced glutathione (GSH) and 2-phenyl-4,4,5,5-tetramethylimidazoline-1-oxyl 3 oxide (PTIO) on larval early 5^th^ instar Heliothis virescens plasma virucidal activity. NAC control (40 mM); NAC+Plasma, NAC (40 mM) mixed with plasma; GSH (10 mM) control; GSH+Plasma, GSH (10 mM) mixed with plasma; PTIO, PTIO control (100 μM); PTIO+Plasma, plasma mixed with 100 μM PTIO. The inhibitors were added to diluted plasma samples before the addition of *Hz*SNPV (letters indicate significant differences between treatments; n = 4, mean ± SEM).

Several reactive free radicals have been detected in the hemolymph of insects in addition to those generated by phenoloxidase (Nappi *et al.* 2004). Activated phenoloxidase generates a toxic cloud of free radicals including cytotoxic quinones and reactive oxygen species (Nappi *et al.* 2004). In order to rule out a contribution of other free radical generating enzymes from consideration, we examined the effect of several free radical scavengers upon plasma virucidal activity (Fig. 3). The free radical scavenger N-acetyl-cysteine inhibited virucidal activity, as did reduced glutathione. Addition of the nitric oxide scavenger PTIO to plasma before addition of HzSNPV had no detectable effect upon virus survival (n = 4, p < 0.011, Student-Newman-Keuls multiple comparison test; Fig. 3).

In separate experiments we had observed that inclusion of excess dietary ascorbic acid inhibited the melanization activity of larval H. virescens plasma. High plasma levels of ascorbic acid might then be expected to alter the melanization-dependent virucidal activity. Larvae were reared on artificial diet supplemented with increasing amounts of ascorbic acid, and pupation, adult emergence and mortality data were collected. The time required for larvae to progress to pupation and adult emergence increased with the concentration of added dietary ascorbic acid (Fig. 4A). Larvae fed basal diet (2.4 mg/ml) attained 50% pupation after 12.8 ± 0.1 days and 50% had emerged as adults by 24.8 ± 0.2 days. When dietary ascorbic acid levels were raised to 12.0 to 48.0 mg/ml (5 to 20 times the basal level) pupation was delayed until 13.2 ± 0.2, 14.8 ± 0.2, and 17.6 ± 0.4 days, while emergence was delayed until 25.3 ± 0.3, 27.3 ± 0.3, and 29.5 ± 0.5 days, with highest mortality at the highest concentration of ascorbic acid (Fig. 4A). Pupal weights did not differ significantly between those reared on diets containing from basal to 24.0 mg/ml ascorbate (246.5 ± 26.5, 247.8 ± 33.4, and 250.7 ± 26.6 mg/pupa) (Fig. 4B). However, the pupal weight attained by larvae reared on diet containing 48.0 mg/ml ascorbic acid was significantly lower than the other groups (220.8 ± 28.2 mg, n = 30, p < 0.018, Dunn’s pairwise multiple comparison test; Fig. 4B) which correlated with the very high mortality of this group (62%).

**Figure 4A.**
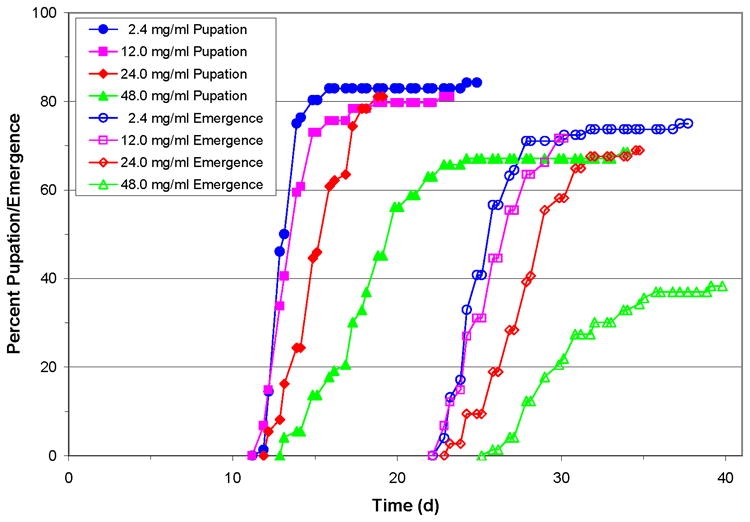
Elevated levels of dietary ascorbic acid resulted in delayed pupation and adult emergence times of Heliothis virescens larvae compared to those reared on basal diet. Diets were supplemented with 12.0, 24.0, and 48.0 mg/ml ascorbic acid (5 to 20 times the basal level (2.4 mg/ml) of ascorbic acid. Larvae were placed on the diets as neonates (n = 75).

**Figure 4B.**
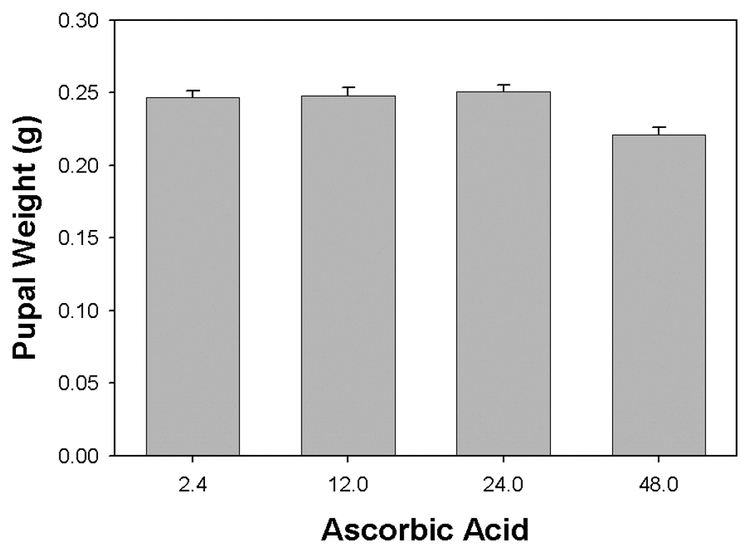
Elevated levels of dietary ascorbic acid resulted in reduced pupal weights attained by Heliothis virescens larvae compared to those reared on basal diet. Basal diet was supplemented with 12.0, 24.0 and 48.0 mg/ml ascorbic acid (basal level 2.4 mg/ml). Larvae were placed on the diets as neonates (letters indicate significant differences between treatments; n = 30, mean ± SEM).

Plasma collected from early 5^th^ instar H. virescens larvae reared on basal diet, and from larvae reared on diets supplemented with from 4.8 to 14.4 mg/ml ascorbic acid, did not exhibit significant inhibition of virucidal activity (n = 4, p < 0.001, Dunn’s method multiple comparison test; Fig. 5A). However, plasma collected from larvae reared on diets with from 24.0 to 48.0 mg/ml ascorbic acid contained no residual virucidal activity (Fig. 5A). Because high dietary levels of ascorbic acid may result in higher plasma concentrations of ascorbic acid it is probable that HvPO activity was reduced in these larvae. We did not measure plasma ascorbic acid concentrations following dietary supplementation. However, in support of the hypothesis, aliquots of the diluted plasma collected from larvae reared on the 24.0 and 48.0 mg/ml ascorbic acid diets failed to melanize. Aliquots of plasma collected from basal diet fed larvae, and 4.8 to 14.4 mg/ml ascorbic acid fed larvae did visibly melanize. Visible melanization of plasma aliquots correlated very strongly with plasma virucidal activity. Addition of increasing concentrations of ascorbic acid directly to plasma from larvae reared on basal diet exhibited a similar pattern of inhibition of virucidal activity (Fig. 5B). *In vitro*, virucidal activity was completely inhibited at concentrations of 10 and 50 mg/ml (n = 4, p < 0.002, Student-Newman-Keuls multiple comparison test; Fig. 5B).

**Figure 5A.**
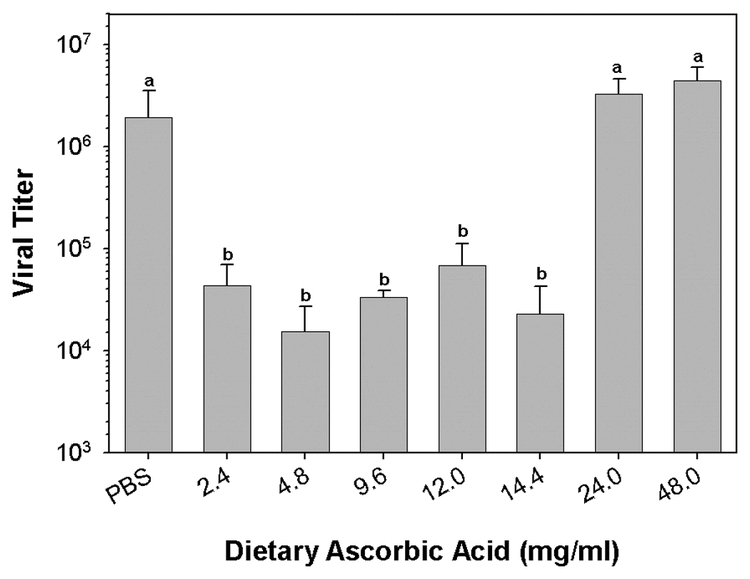
Elevated levels of dietary ascorbic acid inhibit 5^th^ instar larvalHeliothis virescens plasma virucidal activity. Larvae were reared on basal diet from the neonate stage, and on diets supplemented with 4.8 to 48.0 mg/ml ascorbic acid (basal level 2.4 mg/ml). Plasma was collected from early 5^th^ instars for assay of virucidal activity (letters indicate significant differences between treatments; n = 4, mean ± SEM).

**Figure 5B.**
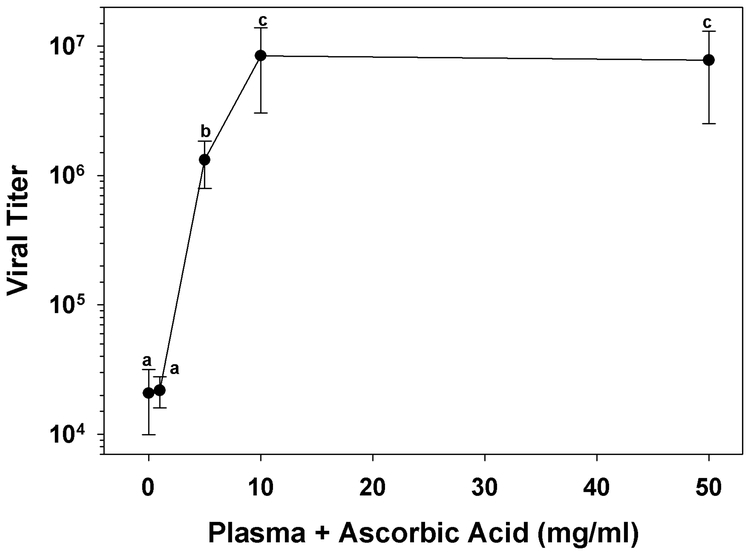
Ascorbic acid inhibits the *in vitro* plasma virucidal activity present in 5^th^ instar larval Heliothis virescens HzSNPV. Following a 1 hr incubation with HzSNPV plasma was assayed for virucidal activity (letters indicate significant differences between treatments; n = 4, mean ± SEM).

**Figure 6.**
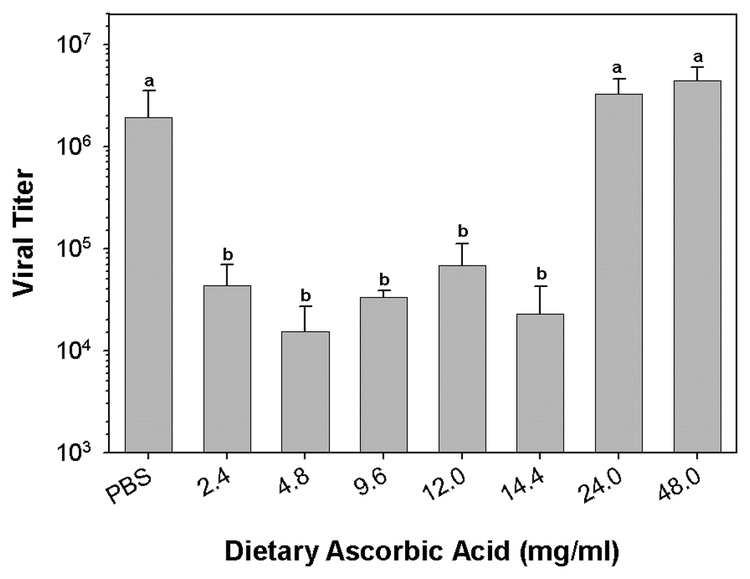
Enzymatic scavenging of the reactive oxygen species H_2_O_2_ from early 5^th^ instar larvalHeliothis virescens plasma reduces virucidal activity. PBS, No plasma control; Cat, catalase (100 U/ml) control; Cat+Plasma, catalase incubated with plasma; superoxide dismutase (100 U/ml) control; superoxide dismutase +Plasma, incubation of plasma with the enzyme superoxide dismutase (100 U/ml). The enzymes were added to diluted plasma samples before the addition of*Hz*SNPV (letters indicate significant differences between treatments; n = 4, mean ± SEM).

Addition of the enzyme catalase, which inactivates H_2_O_2_, to diluted H. virescens plasma partially inhibited the virucidal activity (n = 4, p < 0.013, Student-Newman-Keuls multiple comparison test; Fig. 6). Addition of the same amount of catalase to *Hz*AM-1 cells had no effect on the survival of added *Hz*SNPV. Addition of the enzyme superoxide dismutase, which inactivates superoxide, had no effect on virucidal activity. Addition of superoxide dismutase to *Hz*AM-1 also had no effect on virus survival. This suggests that at least some of the virucidal enzymatic free radicals generated by HvPO may be H_2_O_2_.

## DISCUSSION

A number of observations converge upon phenoloxidase activity as an important determinant of resistance to entomopathogens. Density-dependent prophylaxis against a wide variety of microbial and viral pathogens observed in lepidopteran larvae is directly correlated with elevated cuticular and plasma melanizing capability,*i.e.* phenoloxidase activity (Wilson *et al.* 2001). Conversely, reduction of plasma melanizing activity, by targeting prophenoloxidase expression in the mosquito Armigeres subalbatus with a Sindbis virus encoding an antisense transcript, eliminated melanization ofDirofilaria immitis microfilaria (Shiao *et al.* 2001). Inhibition of the host melanization response by parasitoids, filarial parasites, bacteria, as well as by entomopathogenic fungi appears to be abetted*via* reduced phenoloxidase activity (Beerntsen *et al.* 2000; Shelby *et al.* 2000). Plasma of larval H. virescens, has been demonstrated to exhibit antiviral activity against several vertebrate viruses *in vitro* (herpes simplex virus types 1 and 2, vesicular stomatitis virus, parainfluenza-3, coxsackie B3, Sindbis virus, and HIV-1) (Ourth and Renis 1993;Ourth 2004) and in addition inactivates the baculovirus HzSNPV (Popham *et al.* 2004). Susceptibility of Helicoverpa zea larvae to AcMNPV infection was elevated by addition of the phenoloxidase-inhibiting copper chelators phenylthiourea and diethyldithiocarbamic to the diet (Washburn *et al.* 1996).

Susceptibility of larval lepidoptera to fatal baculovirus infection generally decreases during development, a phenomenon known as developmental resistance (Hoover *et al.* 2002; Kirkpatrick *et al.* 1998). Instar specific and intrastadial differences in the sloughing of infected midgut cells appears to be the primary mode of resistance to AcMNPV infection of H. virescens larvae (Kirkpatrick*et al.* 1998; Engelhard and Volkman 1995). Intrastadial resistance or variable susceptibility within instars may occur. Mortality following LdMNPV infection of gypsy moth, Lymantria dispar, 4^th^ instar larvae exhibited a biphasic pattern with mortality greatest at the beginning and at the end of the instar (Hoover *et al.* 2002). This pattern was obtained both for *per os* infection and intrahaemocoelic injection indicating that resistance factors operating beyond the midgut barrier (*i.e.*, viral clearing from the hemolymph, or cells becoming refractory to infection) were present (Hoover *et al.* 2002; and references therein). Developmental resistance of H. virescens larvae to AcMNPV infection increased during the first 18 hrs of the 4^th^ instar, but decreased thereafter, while intrahaemocoelic injections of virus exhibited no evidence of developmental resistance (Kirkpatrick et al. 1998).

We have previously reported the inhibitory effects of phenylthiourea and serine proteinase inhibitors on the *in vitro* virucidal activity ofH. virescens larval plasma against HzSNPV (Popham *et al.* 2004). Using the fungal metabolite Kojic acid as a specific competitive inhibitor of insect phenoloxidases (Dowd 1988; Chen *et al.* 1991a; Chen *et al.* 1991b; Li and Kubo 2004) we found that the virucidal activity present in early 5^th^ instar H. virescens larval plasma is dependent upon HvPO activity. These data in combination provide strong evidence that HvPO has a strong virucidal activity against the budded form of baculoviruses.

Generation of cytotoxic free radicals (*e.g.*, semiquinones, superoxide, hydrogen peroxide, nitric oxide) in the hemolymph of insects by soluble enzymatic activities and by lipopolysaccharide-stimulated hemocytes are known responses to microbial, filarial and parasitoid infection (Nappi *et al.* 2004; Nappi and Christenson 2005). In the larval waxworm, Galleria mellonella, phenoloxidase-dependent production of DOPA-semiquinone free radicals is inhibited by fungal infection (Slepneva*et al.* 2003). The possible virucidal activity of these free radicals generated in the plasma of infected insects has yet to be adequately explored. Inhibition of H_2_O_2_ generation by inclusion of the enzyme catalase in the *in vitro* reaction substantially reduced activity. H_2_O_2_ is a known byproduct of phenoloxidase activity (Komarov *et al.* 2005), and this suggests that free radicals produced by phenoloxidase cause viral inactivation. A superoxide- and H_2_O_2_-generating antimicrobial adduct of glutathione and β-alanyl-DOPA (N-β-Alanyl-5-S-glutathonyl-3,4-dihydroxyphenylalanine) was isolated from immunized flesh flies, Sarcophaga peregrina, and also is a byproduct of melanization (Leem*et al.* 1996). Plasma ascorbic acid acts as a free radical scavenger. Excess dietary ascorbic acid suppressed hemolymph melanization, and reduced the melanization of Sephadex beads or Plasmodium berghei by Anopheles gambiae (Kumar *et al.* 2003). WhenH. virescens larvae were reared on diet containing excess dietary ascorbic acid plasma melanization and the virucidal activity were completely inhibited. Thus, the free radical scavengers ascorbic acid, N-acetyl cysteine, and reduced glutathione, all of which block larval H. virescens plasma melanization, also block plasma virucidal activity against HzSNPV.

Nitric oxide inhibits replication of viruses within infected vertebrate cells, and acts against a number of parasitic infestations in a variety of vertebrate and invertebrate model systems *in vitro* and *in vivo* (Nappi *et al.* 2004; Foley and O’Farrell 2003; Ascenzi and Gradoni 2002; Luckhart *et al.* 1998). By including the efficient nitric oxide scavenger, PTIO, in the virucidal activity assay we found that nitric oxide generation does not play a detectable role against HzSNPV *in vitro*. Although our initial results indicate that nitric oxide generation does not account for any significant virucidal activity in larval plasma, the possibility that generation of nitric oxide by other tissues such as hemocytes or fat body, which were excluded from our analysis, are involved in immune reactions cannot be excluded (Nappi *et al.* 2004).

In summary, specific chemical inhibitors of phenoloxidase, or of prophenoloxidase activation, and addition of the enzyme substrate dopamine, provide strong evidence that a constitutive humoral innate antiviral immune response attributable to the activity of phenoloxidase is capable of limiting baculovirus infection beyond the midgut barrier in lepidopteran larvae. Taken together these data establish that products of the plasma enzyme phenoloxidase, including superoxide, contribute to the observed virucidal activity against baculoviruses, and against other classes of viruses. If plasma phenoloxidase constitutes an innate immune response against viral infection *in vivo* then foliar compounds which inhibit phenoloxidase activity may also alter observed mortality to microbial infections (Dowd 1988; Dowd 1999; Kubo et al., 2003). For example, foliar phenolics such as tannic, chlorogenic, and caffeic acids have been demonstrated to inhibit melanization and to influence susceptibility to baculovirus infection (Feldman *et al.* 1999; Ali *et al.* 1999; Hoover *et al.* 1998;Hoover *et al.* 2000). Although one mode of action of these inhibitors appears to be within the lumen of the midgut, our data indicate that inhibitory compounds that diffuse from the lumen into the hemolymph may inhibit phenoloxidase activity in plasma, and may thereby alter susceptibility to viral infection. The importance of these results should be weighed in relation to other insect-transmitted viruses that often must traverse the hemocoelic immune barriers of the vector. Therefore plasma phenoloxidase may be involved in vector competence of arthropods that transmit viruses to vertebrates or plants (Medeiros *et al.* 2004;Beerntsen *et al.* 2000).
